# A Robotic grinding station based on an industrial manipulator and vision system

**DOI:** 10.1371/journal.pone.0248993

**Published:** 2021-03-24

**Authors:** Guoyang Wan, Guofeng Wang, Yunsheng Fan

**Affiliations:** Department of Marine Electrical Engineering, Dalian Maritime University, Dalian, China; Universiti Sains Malaysia, MALAYSIA

## Abstract

Due to ever increasing precision and automation demands in robotic grinding, the automatic and robust robotic grinding workstation has become a research hot-spot. This work proposes a grinding workstation constituting of machine vision and an industrial manipulator to solve the difficulty of positioning rough metal cast objects and automatic grinding. Faced with the complex characteristics of industrial environment, such as weak contrast, light nonuniformity and scarcity, a coarse-to-fine two-step localization strategy was used for obtaining the object position. The deep neural network and template matching method were employed for determining the object position precisely in the presence of ambient light. Subsequently, edge extraction and contour fitting techniques were used to measure the position of the contour of the object and to locate the main burr on its surface after eliminating the influence of burr. The grid method was employed for detecting the main burrs, and the offline grinding trajectory of the industrial manipulator was planned with the guidance of the coordinate transformation method. The system greatly improves the automaticity through the entire process of loading, grinding and unloading. It can determine the object position and target the robotic grinding trajectory by the shape of the burr on the surface of an object. The measurements indicate that this system can work stably and efficiently, and the experimental results demonstrate the high accuracy and high efficiency of the proposed method. Meanwhile, it could well overcome the influence of the materials of grinding work pieces, scratch and rust.

## Introduction

Grinding of metal casts has always been a difficult task in industrial applications as it causes a lot of pollution, consumes a large amount of energy, and poses a high risk in processing. With the application of an industrial manipulator grinding station for the grinding process, this situation has been significantly improved. The method of grinding using an industrial manipulator makes full use of the flexibility, high speed and simple programming of the robot [1]. As one of the main sensing technology of robots, machine vision is popular because of its non-contact nature and ability to collect a large amount of information [2]. Vision-based grinding stations can realize automated grinding, reduce labor costs, improve the consistency of the ground objects and optimize and shorten production cycles [3, 4].

However, there are still problems when a manipulator is used to grind metal casts: (1) The feeding process is difficult: the loading of the object is carried out by the operator and fixed to the specific fixture. Following this, the manipulator completes the task of grasping the object. However, the casts are irregular in shape, and the weight of some metal castings is above 20 kg. Thus, the manipulator operation consumes time, and manual operation is not safe; (2) Grasp positioning is difficult: it is necessary to position and grasp the same region on the object’s surface in the same posture each time. Hence, designers need to design special fixtures for each product. When the production line needs to switch to objects of different shapes and volumes, the fixture must be redesigned. This not only consumes labor but also increases the hardware expenditure [5]; (3) The grinding trajectory of the manipulator and the special equipment are complicated [6]: the shape and position of burrs are not certain. It is a common practice to polish the location where burrs may appear on the surface of the object during the grinding process. This increases the working time and workload; (4) Manipulator grinding relies on human teaching: this method is time-consuming and labor-intensive and in order to ensure accuracy, the worker must be close to the equipment while teaching the trajectory. In the process of grinding and debugging, dust and other harmful substances are easily generated, which are harmful to the health of the worker.

To solve above problems, several studies have been undertaken using industrial manipulators for grinding metal castings. Park [7] proposed a method for industrial manipulators to cut big casting objects; however, it is difficult when another object needs to be worked on. The method relies on manual loading and unloading and the processing trajectory relies entirely on manual teaching. In another study, Gaz [8] proposed a system that uses collaboration between robots to polish an object with a human operator working in the same area. The system is safe and flexible; however, the grinding trajectory needs to be taught by a human. There are also many polishing systems that combine a force sensor and an industrial manipulator [9, 10], which can improve the grinding quality and reduce the system working time. But these systems still need substantial involvement of humans during operation. Visual technology is also often used in robotic grinding. An automatic robotic grinding system based on reverse engineering has been realized [11]); however, its trajectory is based only on the surface shape of the product and the burr on the object is not considered. Pandiyan [12] proposed a method based on deep learning techniques for the detection of the weld seam and its removal; however, this method has a high rate of misclassification between the weld seam states and the background.

To address the above issues, we propose an automatic grinding workstation based on an industrial manipulator and vision system which can be used for processing rough metal casts in this study. The system can robustly complete the positioning and grasping of workpieces placed on the pallet without manual intervention and can regularize the robot grinding trajectory according to the shape of the workpiece burr to achieve the automatic operation of the grinding process. A two-step localization strategy has been proposed to obtain the position of the object in the pallet. This strategy has the advantages of excellent generalization performance of the deep neural network and high positioning accuracy of template matching. It can obtain the position of a blank cast object accurately even in the presence of interruptions. Following this, we propose a grid method to detect the burr position and offline planning of the industrial manipulator grinding trajectory. The system can stably and quickly position the objects placed at regular intervals on the pallet. The object’s grinding trajectory can be generated offline. Our system also has an excellent price/performance ratio.

The main contribution of this paper is summarized as follows.

1. An automatic grinding workstation for rough casts is designed. The workstation can plan the grinding path according to the main burrs on the workpiece surface.
2. A strategy for visual detection and positioning for rough casts has been proposed to solve the problem that the vision system has a complicated background and cannot use a large size light source in some industrial area. This strategy includes an improved yolov3 detector, i.e., Den-yolov3 and a modified template matching method. This approach offers favorable performance and fast speed for the identification of industrial objects.
3. A grinding trajectory method for industrial robot based on the grid method is proposed. The automatic grinding path planning of industrial robot based on the vision system is realized.

## Methodology

### Hardware architecture and architecture of the proposed method

The grinding station setup is composed of loading cell, grinding cell, unloading cell:

Loading cell: A 2D industrial camera and light are installed in the 6th axis flange of the loading industrial manipulator. The system realizes the positioning of the workpiece through the vision system, and the industrial manipulator completes the grasping operation.
Grinding cell: It consists of two industrial manipulators and two grinding machines. They work together to finish the grinding operation.
Unloading cell: A manipulator is used to realize the unloading and palletizing work.

**Fig 1:**
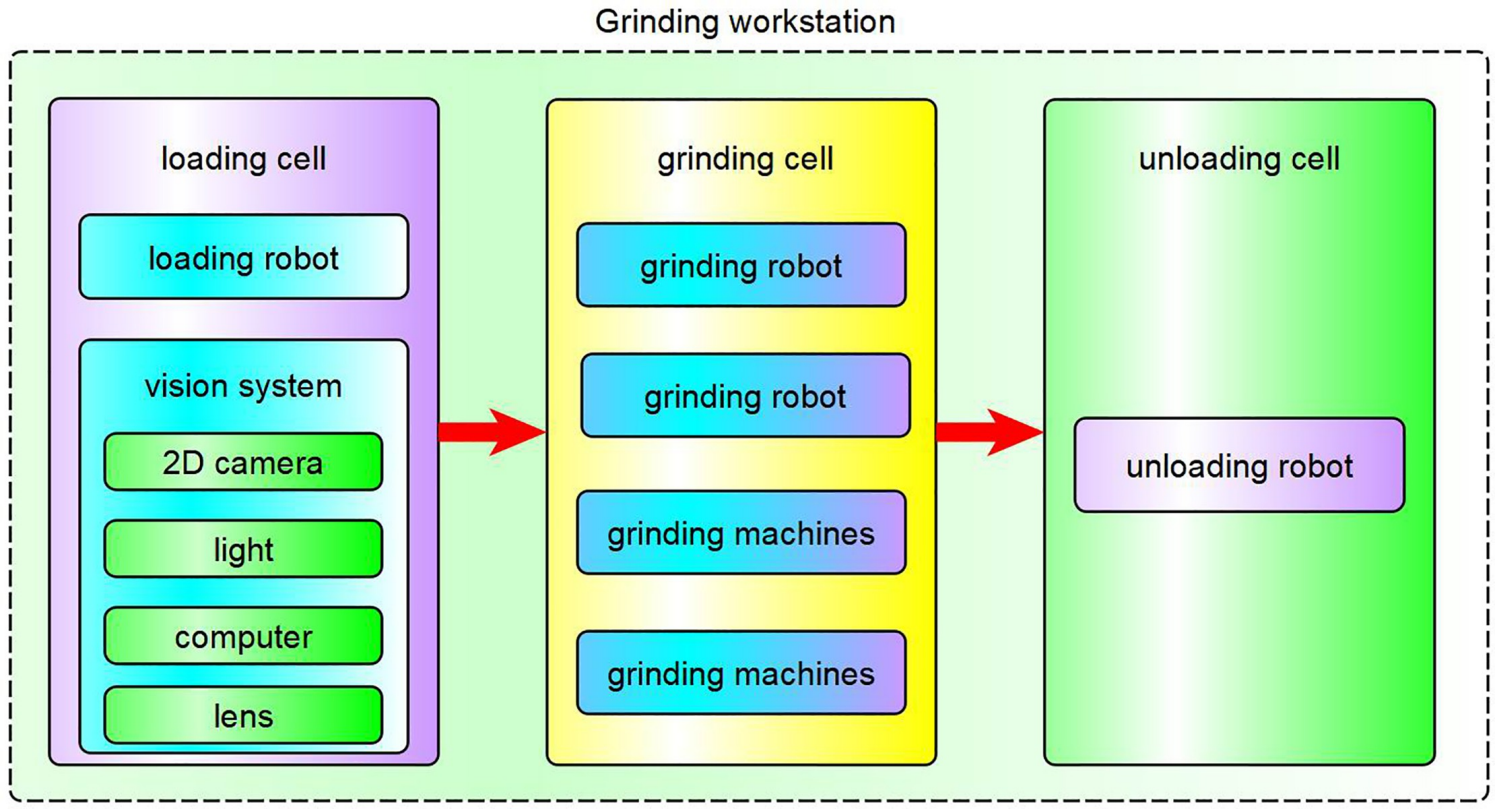
The system architecture of the grinding station.

**Fig 2:**
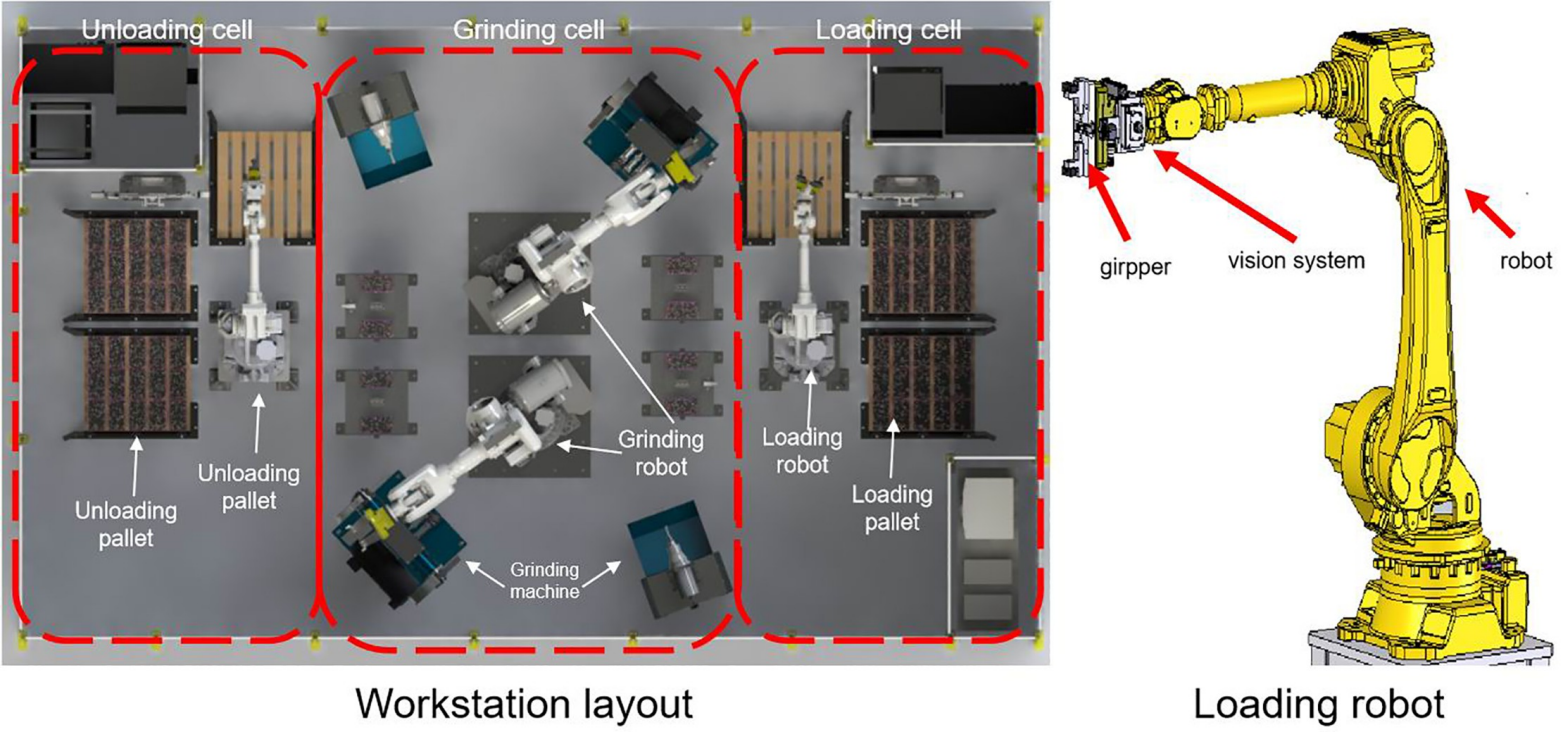
The layout of the grinding station and unloading robot with vision system.

The workstation is controlled by a PLC control system. Figs 1 and 2 show the system architecture and workstation layout, respectively.

The object that is to be processed by the workstation is an iron casting. It is uniformly placed on a pallet with approximately 18 objects per pallet, each with six layers and each separated by a partition. The size of the object to be ground is 290 mm × 155 mm, and its weight is 7 kg. The grinding requirement is that there is no burr around the object’s edges. The object to be ground is shown in Fig 4. During the processing, the staff only needs to place the pallet filled with rough objects in the loading area. The workstation will finish the grinding of the rough objects without manual intervention and place the processed objects in the unloading pallet. The grinding error in this method does not exceed ±0.5 mm.

**Fig 3:**
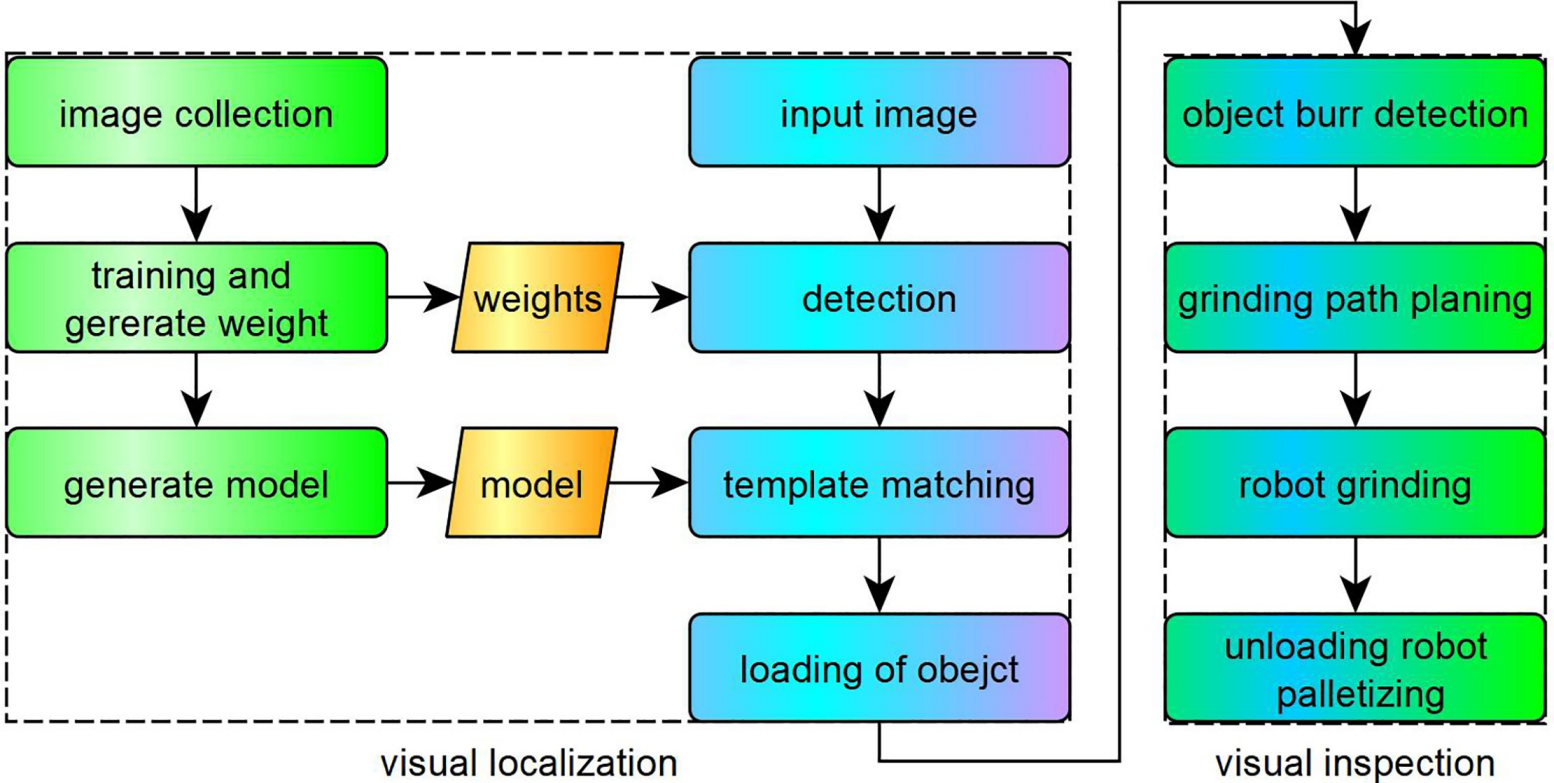
The strategy of robot workstation.

To accurately and efficiently implement object grinding, combined with the hardware system, we designed a workstation automatic grinding strategy, which mainly consists of two parts: visual positioning and visual inspection (see Fig 3).

### Theoretical basis

#### Visual localization

The visual processing of the grinding workstation mainly includes two steps: visual localization during the loading process and burr detection during the grinding process.

**Fig 4:**
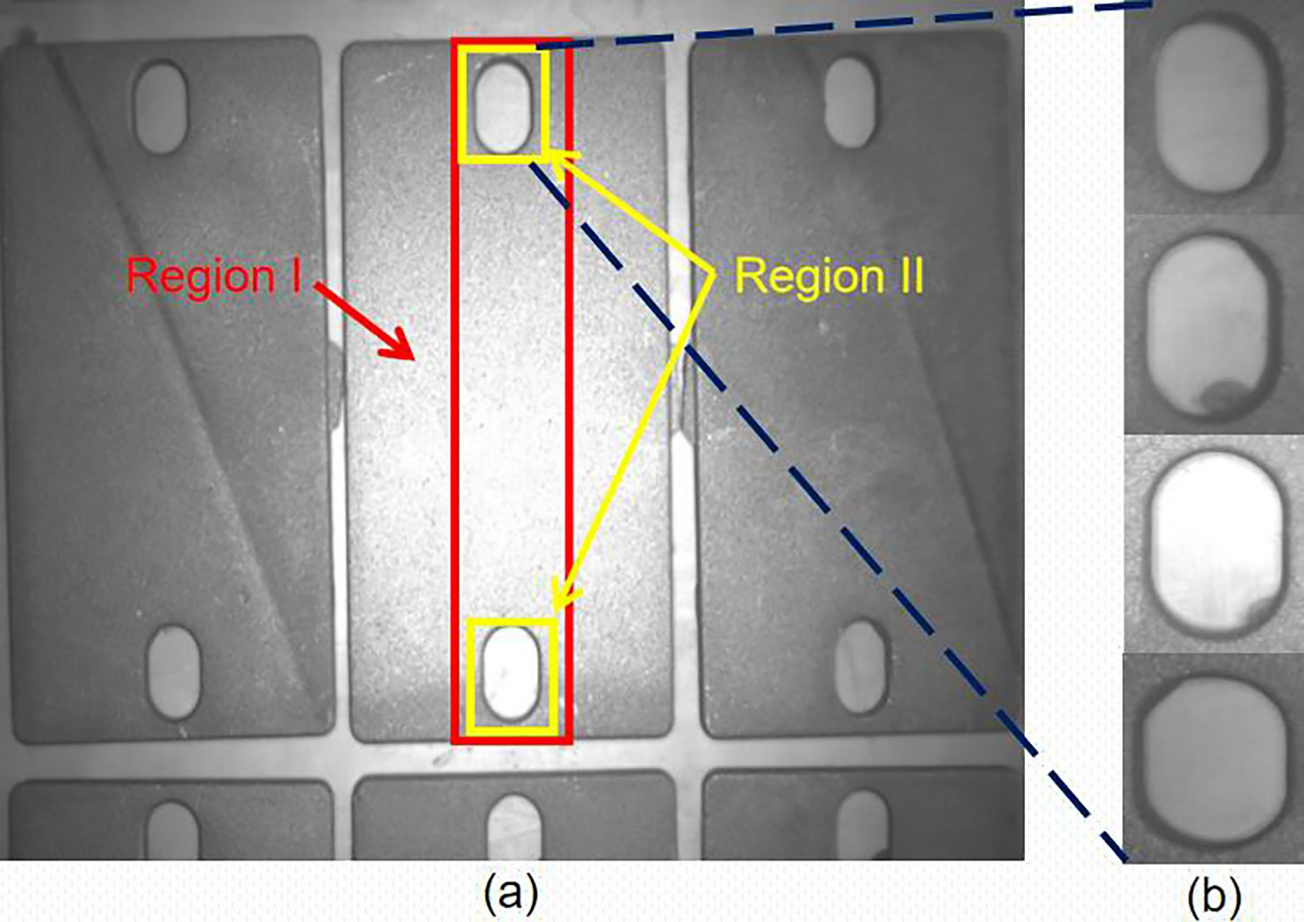
(a) The object and the region used for detection. (b) The contour of the object is not clear in the image.

In this paper, the object has burrs around its edges and the background has the same type of objects near the object that is to be grasped. The most difficult problem that needs to be solved is how to locate an object precisely when it does not have a clear contour and is in a cluttered background (see Fig 4).

A 2D vision system is used to obtain the precise position of the object. In the 2D visual positioning technique, template matching [13] is a commonly used method for positioning. It uses a representative part of the object’s image to be identified as a “model” and uses this “model” to find the object to be identified in the searched image [14]. There are four main reasons why the template matching method is not useful to obtain the object’s position precisely in an industrial area:

1. In the open industrial environment, the image of an object being captured is easy to get influenced by ambient light.
2. The surface of the rough metal casts which is used for image processing has scratches and stains.
3. The burr on the surface of rough metal cast affects the accuracy of the positioning algorithm
4. The rounded transition area of the object surface makes the object contour unstable during the imaging process.

In this paper, we combined the latest deep learning and classic template matching technology to propose a coarse-to-fine visual detection and positioning method. In the coarse positioning stage, we use the deep neural network to detect the approximate position of the workpiece. On this basis, we use the template matching method to precisely locate the local features of the object, thereby achieving the precise positioning of the rough castings with minimal external interference.

*Coarse step*. We use deep learning methods to help make complete use of the features in the detected target. This makes our detection results more robust than a single template matching method.

Deep learning has gradually become the mainstream in target detection after 2012 [15]. At present, many efficient target detection networks have been proposed and applied in the industrial field, such as yolo [16–19], Faster R-CNN [20], and NAS-FPN [21]. The yolo algorithm was proposed by Redmon et al. After two years of development, it has grown from yolo to yolov3. The yolov3 algorithm extracts features based on a regression method. It is an end-to-end training process that directly returns categories and frames on the feature layer, saving a lot of time wasted in extracting frames.

In this paper, we propose an improved yolov3 network for rough positioning of targets.

**Fig 5:**
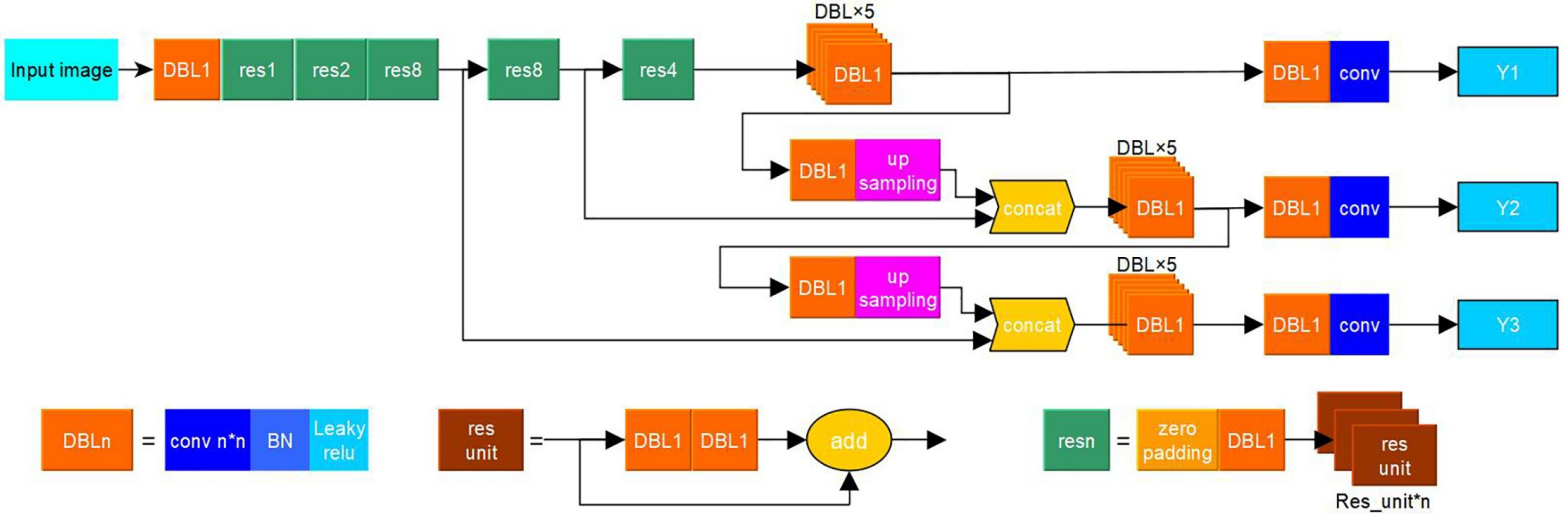
Standard yolov3 network structure.

Standard yolov3 uses Darknet53 network based on residual network structure for feature extraction and generates three feature maps of 13×13, 26×26 and 52×52, respectively (see Fig 5). The three size feature maps correspond to large, medium, and small target detection in the picture.

Our work is mainly focused on the following areas:

1. Set up industrial object image dataset: Through the method of image acquisition and image enhancement, an industrial image-data set containing the object in this study and other industrial object is established.

**Fig 6:**
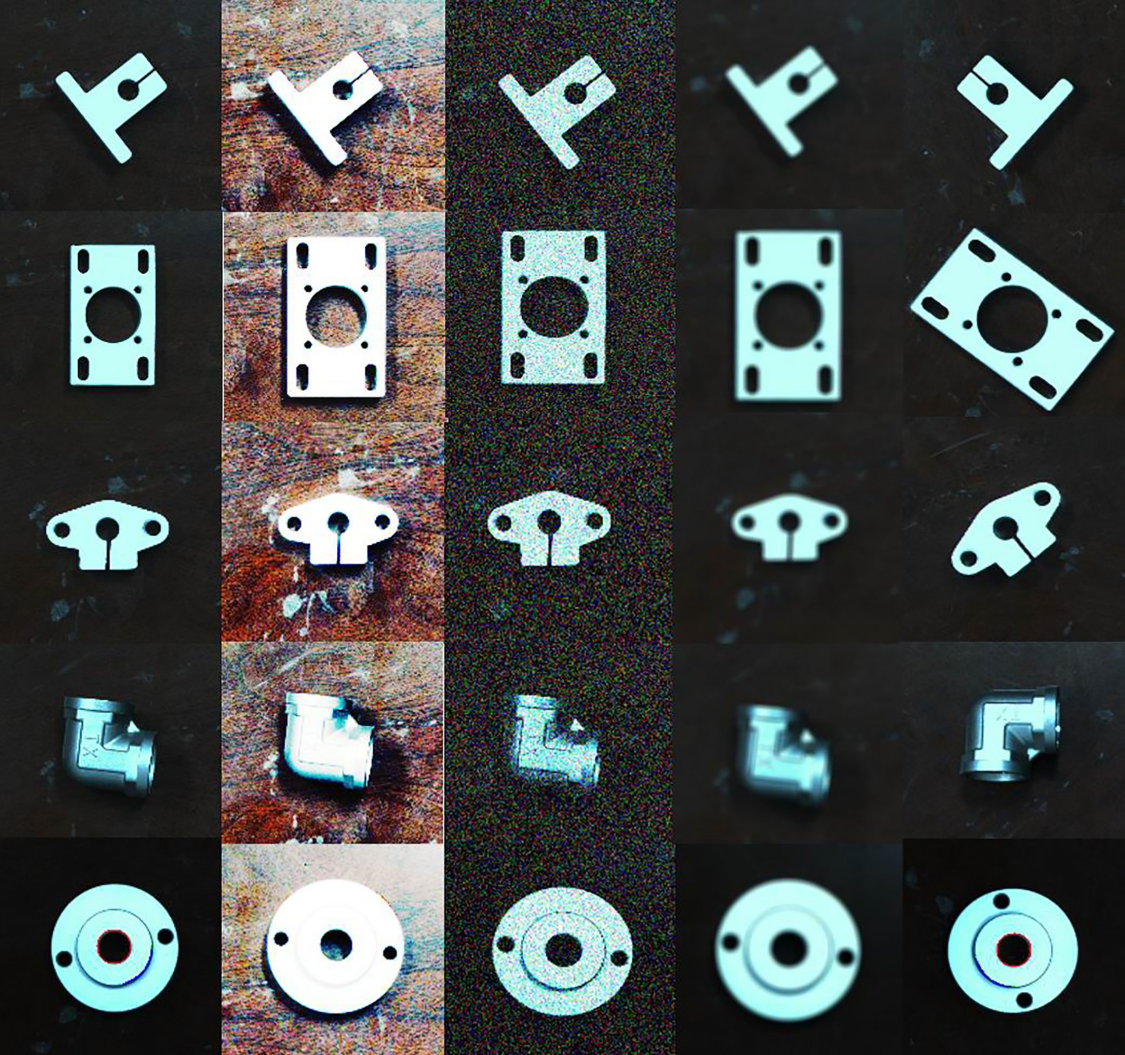
Part of the object in the dataset (from left to right: The original image of the object, image after shading adjustment, the image with Gaussian noise, blurred image, and rotation image).

Image enhancement is an important method to improve the performance of network recognition. Image rotation, adjustment of image brightness, image blur, and image noise are used for data enhancement in this study; an object in dataset is shown in Fig 6.

**Fig 7:**
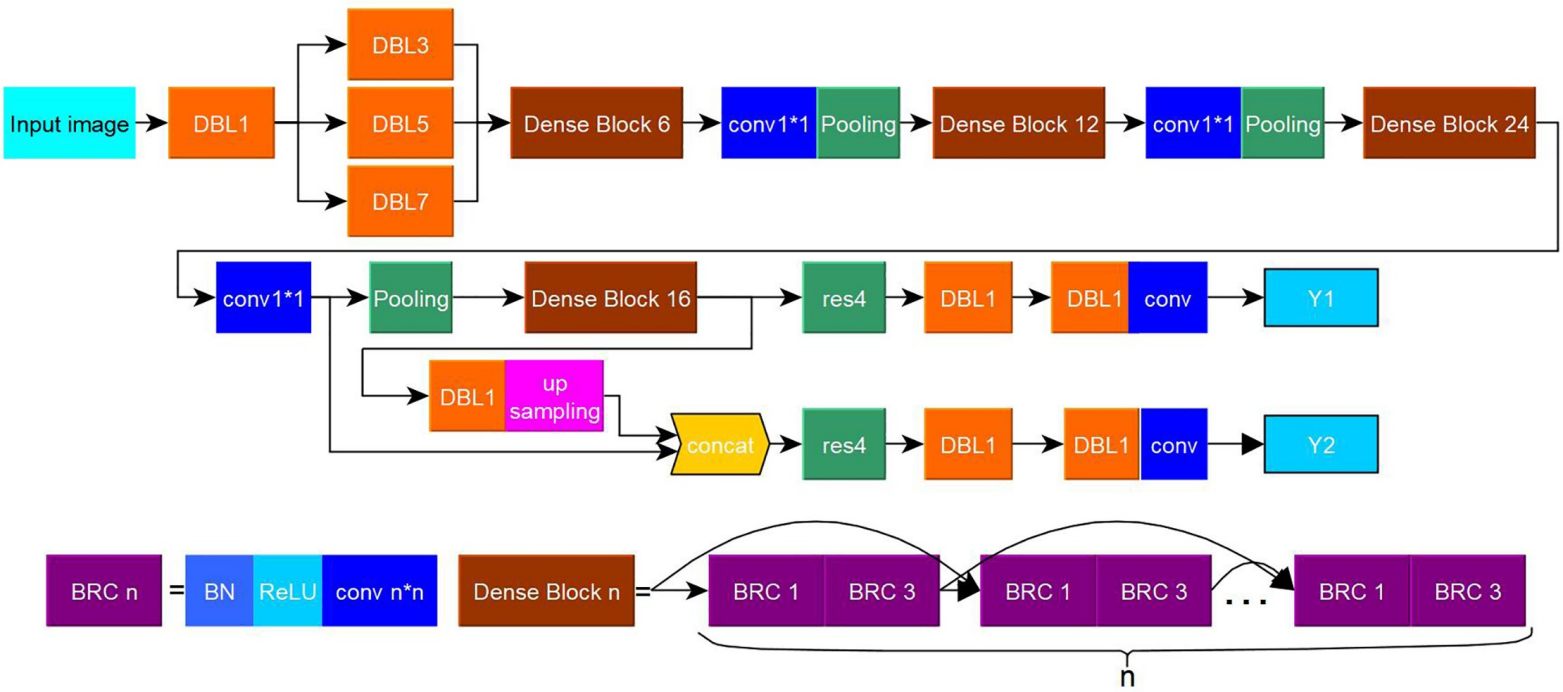
Den-yolov3 network structure.

2. Change yolov3 network structure: 1) The DarkNet network was changed to the DenseNet network, and a parallel network composed of convolution kernel functions of different scales was added to the bottom part of the network, thereby enabling the extraction of richer features in the image. 2) To strengthen the detection of small targets, three size feature maps are used by yolov3 detector for target detection. Since the size of the measured object is not large, the feature map for small targets is removed in this method. 3) The standard yolov3 network uses multiple convolutional networks before outputting the feature map. We introduce the residual structure in this part. The experiments show that the network that introduces the residual structure has better recognition ability. Improved yolov3 is named as Den-yolov3 and shown in Fig 7.

3. Using multiple location features for the object position: To reduce the localization failure caused by recognition errors, we consider multiple local features of the object surface at the same time, and infer the position of the target object according to the detection results of the multiple local features.

Two region features namely region I and region II are select to detect the object in this study (see Fig 4). When the detection is completed, the system calculates the positional relationship between the features in region I and region II to further improve the success rate of detection.

Den-yolov3 can find the positions of all objects in the image, so when the detection is completed, we need to count the position information of the detected regions I and II in the image and select the grasped object.

*Fine step*. After coarse positioning, the approximate location of the object is obtained. We can then determine the approximate location of the circular hole area on the object surface. Localization can be performed in local areas in the picture. Local positioning can improve the robustness of matching and reduce the workload of matching. The Line-MOD method [22, 23] is a contour feature-based template matching method and is one of the most convenient ways to locate objects. Line-2D is a part of Line-MOD with only gradient information from an image. Its excellent and stable characteristics have attracted wide attention from the industry.

We propose the modified Line-2D method, namely, geometric-multilevel-Line-2D (GMLINE-2D), to obtain the object position precisely. The GMLINE-2D method combines geometric model, multilevel matching, and fast ICP registration method into the Line-2D method. It can obtain the object position quickly and robustly. The main step of the GMLINE-2D method is as follow:

The first step of GMLINE-2D is to use the geometric method to create a geometric model for the object feature and generate multiple models map of the geometric model. The feature we selected to create the model is the contour feature of the object feature region I. Unlike the Line-MOD method, which uses the strongest point of the local gradient amplitude as the feature points of the template, the proposed method uses the feature points on the geometric contour of the object surface as the feature points of the template, which can better reflect the contour features of the object.

**Fig 8:**
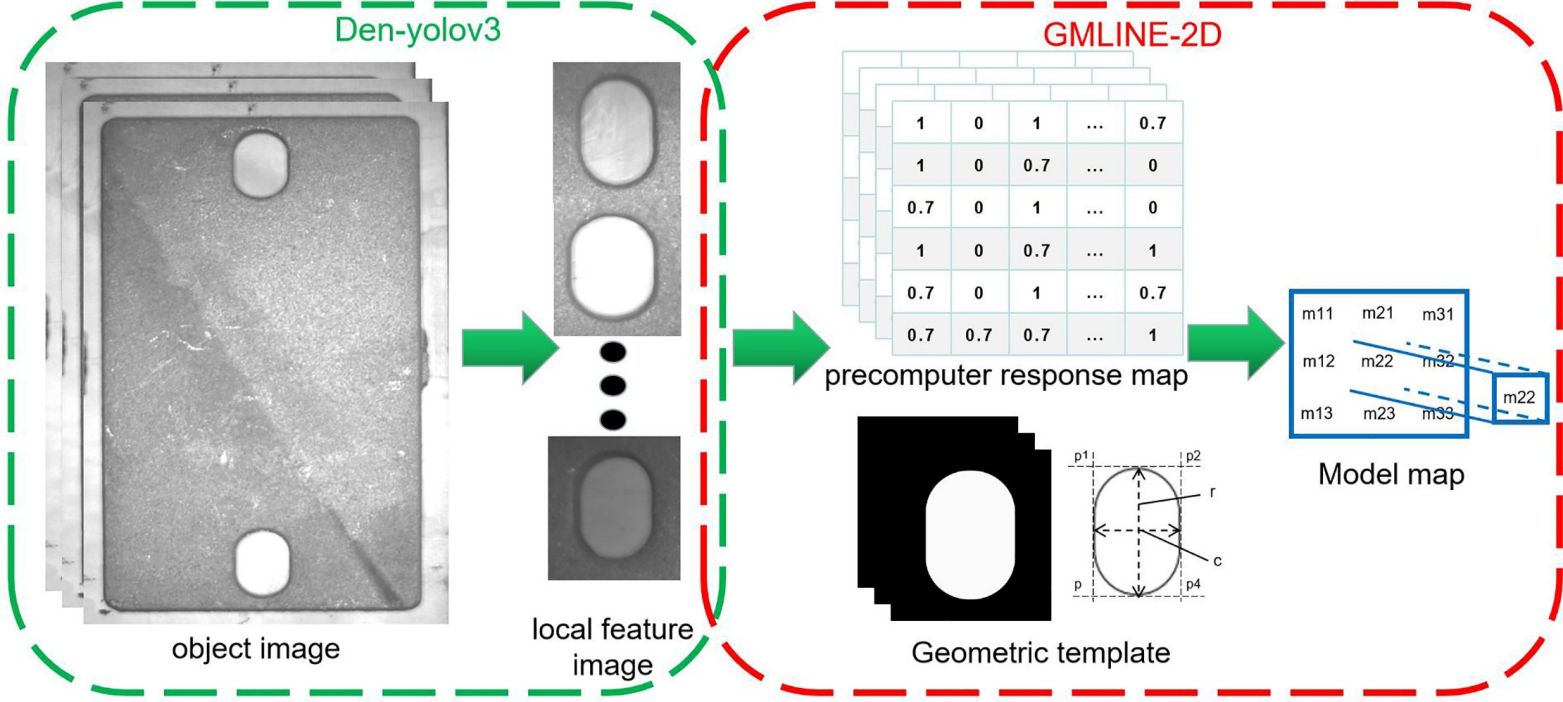
Creating the model map. The model map is created by the local feature of the object. After the local feature is obtained by the Den-yolov3 detector, the geometric method and Line-MOD method is used to generate the model map.

The geometric model includes the main contour information of the object’s feature and it is free of influence by the scratches or stains on the object surface. Thus, this geometric model is robust for template matching. However, because of the height of the object and the influence of ambient light, the object feature in the image shows shape deformation when the object is at a different place in the visual field. To accurately determine the object position, we create multiple models with different shapes to do the template matching and then select the best result. The model map consists of a precomputer response map and multi-scale models; the precomputer response map uses the same step of the Line-mode method [24]. By changing the length and width values of the geometric contour model, several new geometric models can be used to create and generate the models map that consists of multi-scale models (see Fig 8).

The second step is to use a multilevel matching method to obtain the object position. Multilevel matching is a method to improve the speed of template matching. It uses the image pyramid method to build multilevel models. The process is as follows:

Rotate the model m in n degree, and generate *M1* (*m*,…,*m’*) model set. The gradient intensity and angle of the image are calculated. We can get gradient image *G1*, then *M1* is used to match *G1*.

The similarity score is used to measure the similarity between the model and the image to be matched. Generally, the higher the score, the higher the similarity between the model and the input image. In this paper, if the similarity score of the match is greater than 60, the match is successful. We use (4) to calculate the similarity score [24].

$$\varepsilon (I,\;T,\;c)=\sum_{r\in P}\left(\underset{t\in R(c+r)}{\max}\left|\cos(ori(O,r)-ori(I,t))\right|\right)$$ (1)

Among them, *ori*(*O*,*r*) is the gradient of the position in the model. *I* is the image to be matched, and *P* is the set of positions *r* to be calculated in the image *O*, where $R(c+r)=[c+r-\frac{T}{2},c+r+\frac{T}{2}]\times [c+r-\frac{T}{2},c+r+\frac{T}{2}]$ defines the neighborhood of size *T* centered at location *c*+*r* in the input image.

We use the Gaussian pyramid to sample *G1* and the model, to get *G2* and *m2*, template *m2* is rotated at n * 2 degrees and the model set *M2* is generated. Further, the LINE-2D method is used to match *M2* with *G2*.

If the matching score in the previous step is greater than 60, the previous step is repeated.

Through the above steps, we can get the multilevel model of the image. Although the multilevel model is complex in the template establishment process, it can greatly improve the matching speed and stability in the matching process. The actual measurement can carry out four times of downsampling.

**Fig 9:**
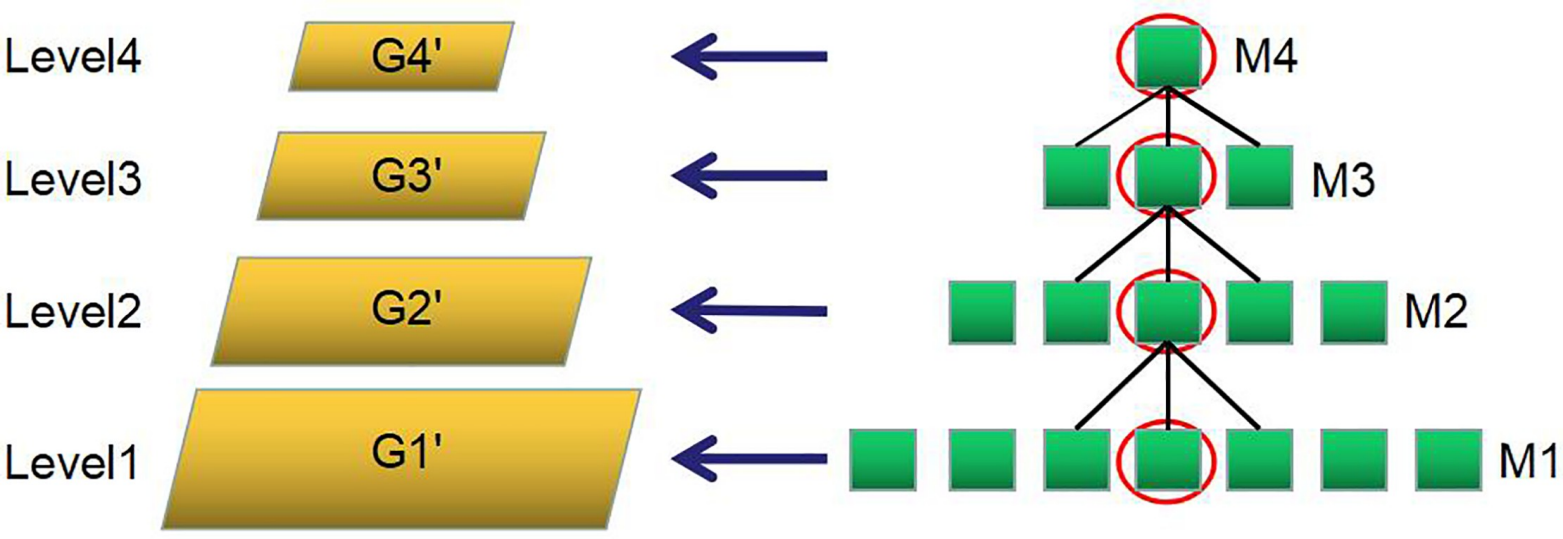
Multilevel matching method (the input image is downsampled to level 4, then M4 set is used for template matching. This determines the M3 model to be used and matches again until the level1).

When we begin multilevel matching, the gradient intensity of the matched image needs to be obtained, and k is used to filter the image, then the Gauss pyramid is used to generate all levels of the image to be matched. It matches with the corresponding template set from the high level (see Fig 9).

After the first level matching template is obtained, the input image needs to be matched again with the model, which is adjacent to the model map. Thus, we can obtain a model whose shape is most similar to the object feature shape in the image.

The last step is to use the fast ICP algorithm to calculate the difference between the location template matching result and the contour of the object in the matched image.

#### Visual inspection

In this work, an offline vision-based robotic trajectory generation system has been proposed. By using the visual technique, the offline trajectory planning of the industrial manipulator is performed according to the shape of the burr in each object to be ground.

*Detecting the position of the main burrs*. There are two kinds of burrs for the object used in the present work, burrs of the sprue gate, generated by demolding and sprue line burrs, generated by the flashing of the sprue line during the casting process.

*Burr position analysis*. Different abrasive tools may have sprue gates located at different positions. However, they are located only in specific areas on the object’s surface, and thus, the burr of the sprue gate is located only at certain specific locations on the object. On the other hand, a flash burr is caused by the aging of the mold, etc., and it may appear on the mold sprue line of the object. Sprue gate burrs are difficult to grind because of their big size. The focus of our work is on how to efficiently grind the sprue gate burrs.

Vision system detection can be used for locating the sprue gate burr position. The detection steps are as follows:

1. After localization, the object has to be placed in a fixture by the industrial manipulator in the presence of ambient light following which, the contour image of the object is obtained by the vision system.
2. Multiple isometric lines are used to calculate the gradient of the object’s edge. We can obtain the edge of the object precisely even though there are flash burrs on the edges of the object.

The sprue gate burrs are located only at specific positions on the object as the shape of the injection mold is fixed. After obtaining the four edge lines of the object by combining this information with the CAD model of the object, we can obtain the possible region of sprue gate burrs and calculate its grayscale value. By checking the grayscale value of the possible region of the sprue gate, we can ensure the position of the sprue gate burrs.

*Burr shape detection*. After obtaining the positions of the sprue gate burrs, we need to detect its shape.

The grinding process investigated in the present work uses an industrial manipulator to grasp the object and grind it using a grinding machine. We set the industrial manipulator to grinding with the same amount of grinding feed. Before grinding, a manual test is done to determine the amount of feed, *M*, per grinding cycle.

**Fig 10:**
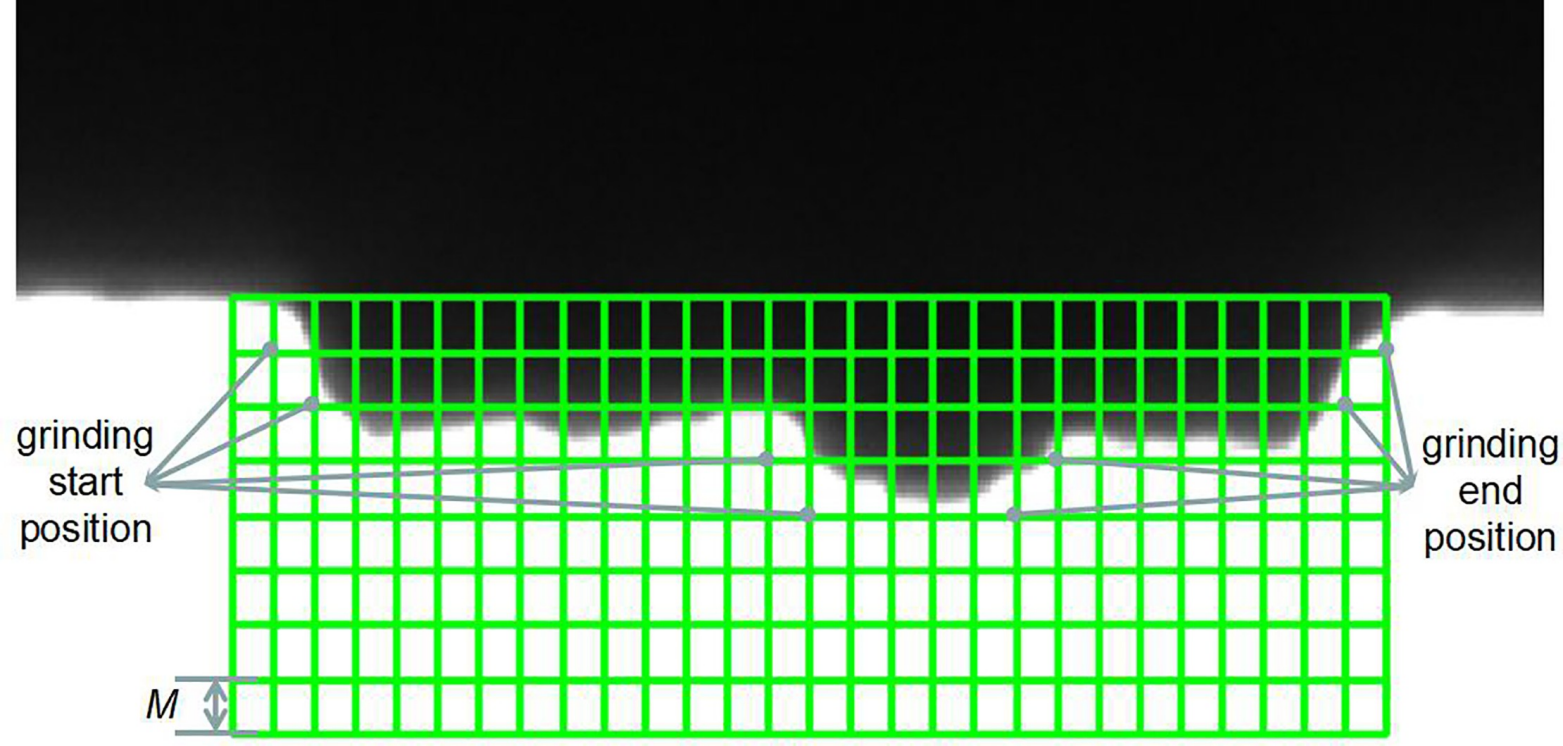
The mesh region method is used to segment the burr area of the casting mouth (M is the amount of feed, it calculate by manual test).

First, the grid method is used to check the contour of the object:

1. Meshing: The Zhang’s calibration method is used to obtain the conversion relationship between pixels and the actual distance [25]. A parallel grid covering a large number of lengths and widths is formed to cover the casting port at the edge of the object, and the side length, *L*, of the mesh is equal to the feed amount *M* of the industrial manipulator grinding track. The system detects the sum of the grayscale values, *W (x*, *y)*, of each line of the grid and then determines whether the burr is included in the mesh area or not.
  W(x,y)<V1;(burrarea)
  V1<W(x,y)<V2;(burredgearea) (2)
  W(x,y)>V2;(noburrarea)
  *V1* and *V2* are the grayscale threshold of the mesh area, it need to be adjusted according to the actual situation.
2. Burr position analysis: The system divides each grid into two categories: to be ground and not to be ground. For example, if a section of the grid does not have a burr, there is no need to polish that area and vice versa. Each row of the grinding area in the grid should have a grinding start and a grinding end position. The starting position is the coordinates of the lower right corner on the grid of the first grid burr area, corresponding to the first burr boundary area in the grid, in each row of the robot grinding direction. The end position is the grid of the last grid burr border area of each line without the presence of burr (see Fig 10).

The grinding start and end positions are the key points of the object to be ground. The value of the key point *P{p1*, *p2*,…, *pn}* of the area to be ground, on the surface of the object in the image coordinate system, can be obtained. We can obtain the burr shape from the key points of the area. In the next step, the grinding trajectory for grinding the burrs can be calculated from the key points.

Vision-based grinding trajectory generation: Using the camera calibration results, the visual coordinate system *{C}* can be obtained. Using the straight-line extraction method combined with RANSAC, the straight line of the two adjacent edges of the object can be accurately obtained from which the intersection point is obtained. This point is taken as the origin to establish the object coordinate system *{O}*.

A user coordinate system *{U}* can be created using the grinding wheel’s grinding position *P*. The positions in *{U}* in the industrial manipulator base coordinate system *{B}* are ^B^*T*_*P*_.

The object is grasped by the industrial manipulator and placed at the corner point *C* of the object (i.e., the origin of the object coordinate system *{O}*) horizontally at the point *P*. The values *{T0}* of the coordinate system ^B^*T*_F_, are taught and recorded

$${}^{B}T_{P}{=}^{B}T_{F}\times^{F}T_{C}$$ (3)

^B^*T*_P_ is the point *P* in the pose of an industrial manipulator base coordinate system. In the grinding process, *C* is a point on the surface of the object which moves as the object moves and ^F^*T*_C_ is a point in *{T0}* coordinate system. Thus, there are:

$${}^{F}T_{C}{=}^{F}T_{O}\times^{O}T_{C}$$ (4)

We have:

$${}^{F}T_{O}\times^{O}T_{C}{=}^{B}T_{F}^{-1}\times^{B}T_{P}$$ (5)

$${}^{F}T_{o}={\left[{}^{B}T_{F}\right]}^{-1}\times^{B}T_{P}\times {\left[{}^{O}T_{C}\right]}^{-1}$$ (6)

^*O*^*T*_*C*_ is the point *C* in *{O}* coordinate system, it can be obtained from the vision system coordinate system. When grinding point position *C* is changed to *C1*, we have:

$${}^{F}T_{C1}^{2}{=}^{F}T_{O}\times^{O}T_{C1}$$ (7)

Combined with (7), there are

BTF'=BTP×[FTO×OTC1]−1 (8)

From this, the new ^B^*T*_F_’ can be obtained, which is the grinding position of the point *C1* of the object.

By doing the coordinate transformation, the object’s grinding key point can be transformed into the position that can be executed by the industrial manipulator.

The visual inspection process generates an offline robotic trajectory based on the shape of the burr in the object and thus replaces the traditional manual teaching method. The offline robotic trajectory generation method can improve the production efficiency, reduce the robot trajectory teaching time of the operators, and enable greater system safety.

## Results and discussion

### Experimental equipment

**Fig 11:**
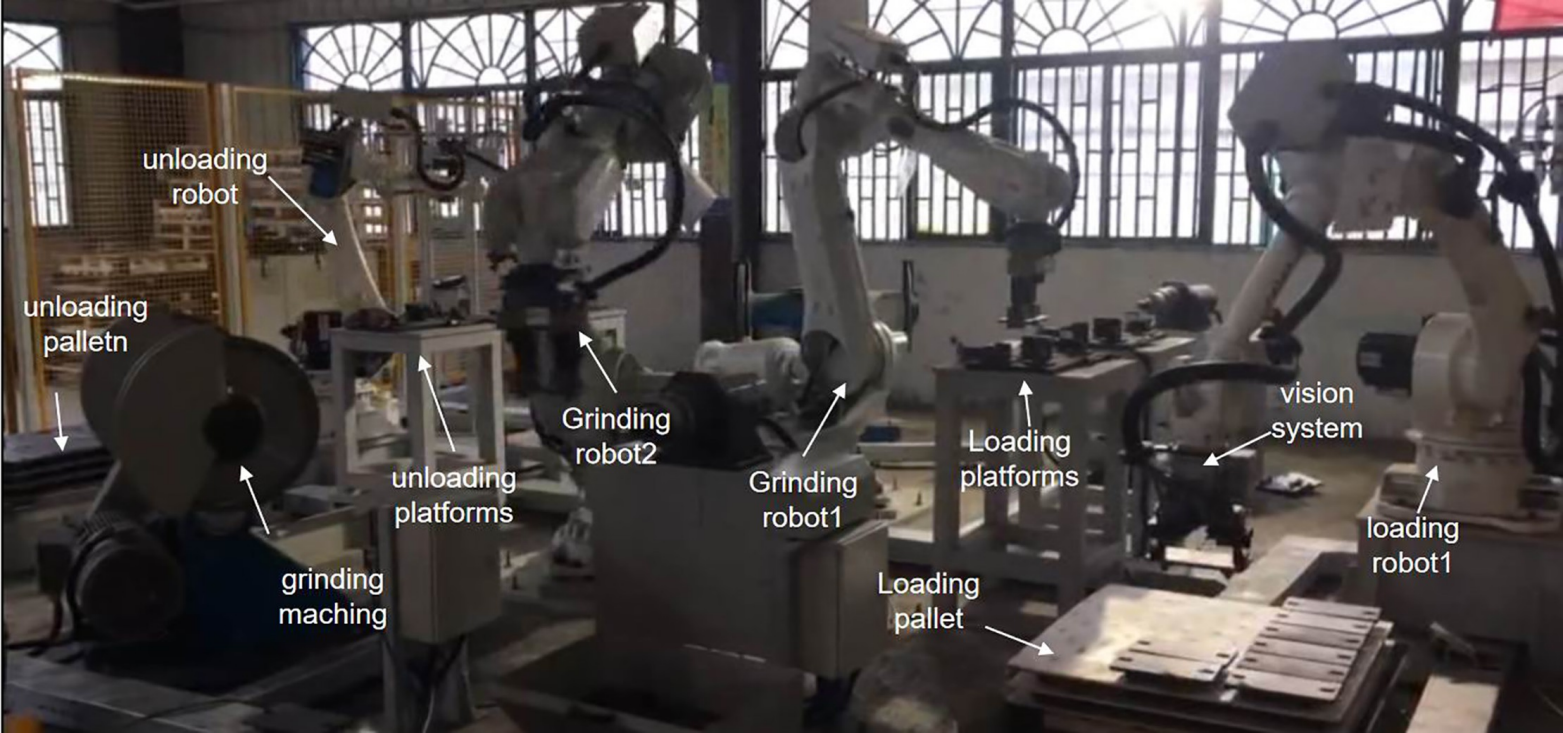
Picture showing the grinding system.

In the system, two EFORT ER50-C10 industrial manipulators, having a payload of 50 kg, were used for loading and unloading and an ABB IRB6700 series industrial manipulator, having a payload of 150 kg, was used for grinding. A vision system (including one Basler ace–3800 series camera and a 12 mm Basler lens) was mounted at the end of the 6th axis flange of the loading industrial manipulator. It is to be used for obtaining the position of the object in the pallet and feed it into loading platforms. The resolution of the camera is 3840×2748, the working distance of the vision system is about 700mm, and the field of vision is about 500mm * 300mm. Two grinding robots take the object from the loading platforms, respectively and grind it on the grinding machine. After grinding, the two grinding robots respectively take parts from the loading table and finish grinding on the grinding machine. After that, the objects are placed on the unloading platforms, and the unloading robot completes palletizing. The PC workstation used in the test with CPU:i7, GPU:RTX 2070S, RAM:32G. C++ language and opencv libraries is used to programming. Two grinding machines grasp the object from loading platforms, then use it for grinding. The experimental setup is shown in Fig 11.

### Coarse positioning experimental

The dataset which we build in this study includes the training data and test data. The training data set consists of the original image and the enhanced image, and the testing data contain 200 original image of different objects. The training data are used to train the stand yolov3, and Den-yolov3 models and test data are used to test the model function. The training data and the main initialization parameters are shown in Tables 1 and 2. The testing data sets are used to detect network performance, the result is shown in Table 3.

**Table 1:** Training dataset.

Index	Image augmentation	Number
1	Object types	6
2	Original image	300
3	Image rotation	5400
4	Brightness transformation	600
5	Image blur	300
6	Gaussian noise	300
7	Total image	6900

**Table 2:** Initialization parameters of the two networks.

Index	Parameters	Value
1	Decay	0.0005
2	Momentum	0.9
3	Learning rate	0.0001
4	Batch	6
5	Image size	416*416
6	Subdivision	4

**Table 3:** Test result of stand yolov3 and Den-yolov3.

objects		Stand-yolov3	Den-yolov3
*Object1*	*Recall*	100%	100%
	*Precision*	0.476%	71.42%
*Total object*	*Recall*	0.776	0.712
	*Precision*	0.901%	0.832%

Recall and precision are used to evaluate the detector performance; the recall is expressed as follows:

$$Recall=\frac{TP}{TP+FN}$$ (9)

The precision is expressed as follows:

$$Precision=\frac{TP}{TP+FP}$$ (10)

*TP* is to predict positive class as a positive class number, *FP* is to predict negative classes to positive classes, and *FN* is to predict positive classes to negative classes. Both stand-yolov3 and Den-yolov3 are trained 300 times. From Table 3 we can find that the recalls of the two detectors are the same, but Den-yolov3 shows higher precision.

FPS reflects the detection speed of the network. The Table 4 shows the size and FPS of stand-yolov3 and Den-yolov3. Form Table 4, we can see that Den-yolov3 has better detection speed and smaller size than stand yolov3. This is because Den-yolov3 removes the small target detection channel in yolov3, which makes it have a smaller size model and faster detection speed.

**Table 4:** Initialization parameters of the two networks.

Method	Model size(MB)	FPS
Yolov3	235	14.02
Den-yolov3	104	14.3

To determine the object position, we took 300 images of the workpiece to be captured in the working environment and selected 200 as the training set and 100 as the test set. The Den-yolov3 and the standard yolov3 mentioned in this paper were used for comparison. Accuracy is the ratio of the number of correct classifications to the total sample. Table 5 shows the accuracy of the two method for 100 and 300 times of training. From Table 5 we can find that Den-yolov3 is better for the object in this study.

**Table 5:** Accuracy of stand yolov3 and Den-yolov3.

Method	100 times	300times
Stand yolov3(Accuracy)	0.78%	100%
Den-yolov3(Accuracy)	0.99%	100%

**Fig 12:**
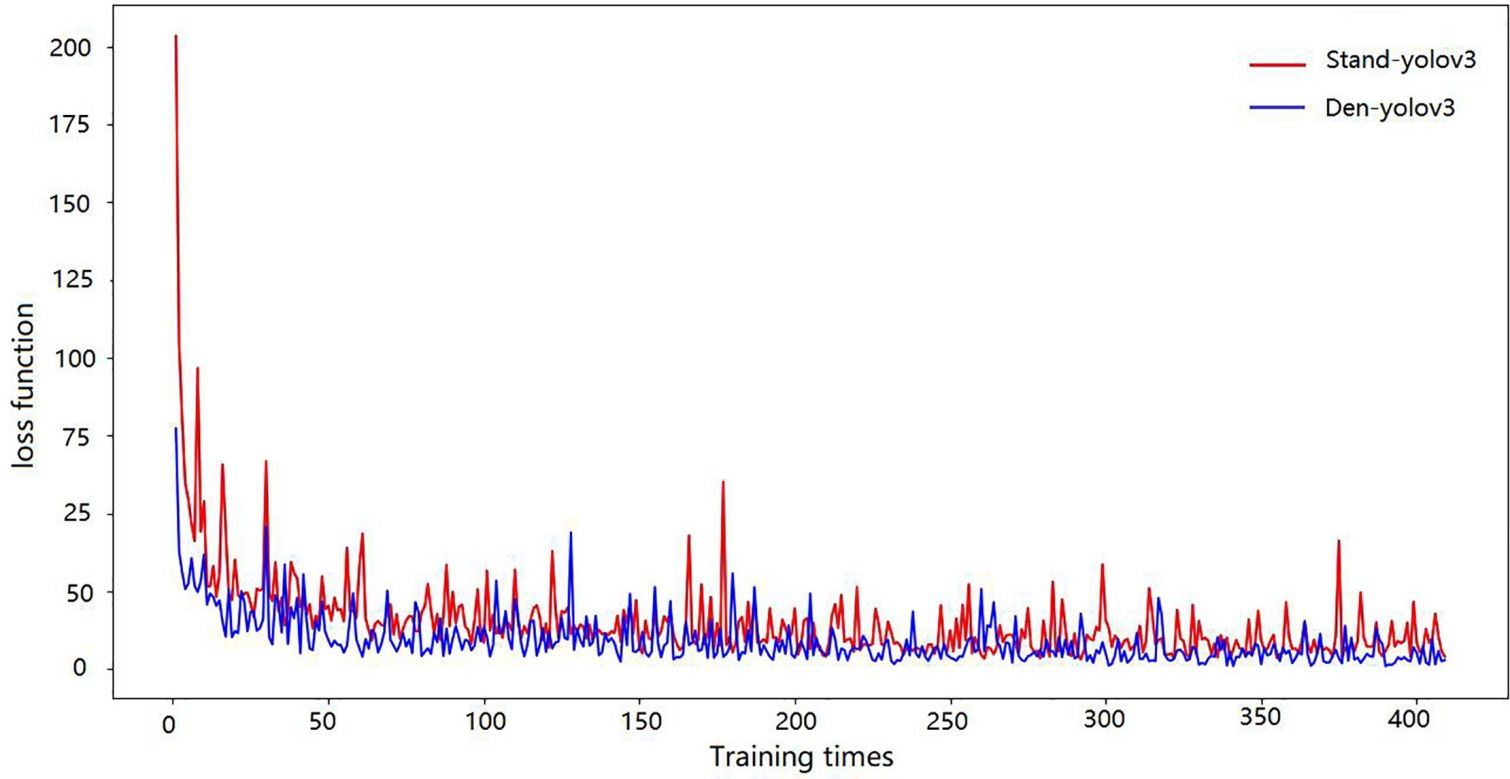
The loss function of stand yolov3 and Den-yolov3.

Fig 12 shows that the loss function of our method decreases rapidly, and the detection success rate is higher under the same training times. This is because, compared with the standard yolov3 algorithm, the Den-yolov3 algorithm has fewer feature maps, so the loss function decreases quickly. The improved network introduces a parallel network, DenseNet network purchase, and residual network, so it can obtain better recognition results under the same iterative training situation.

**Fig 13:**
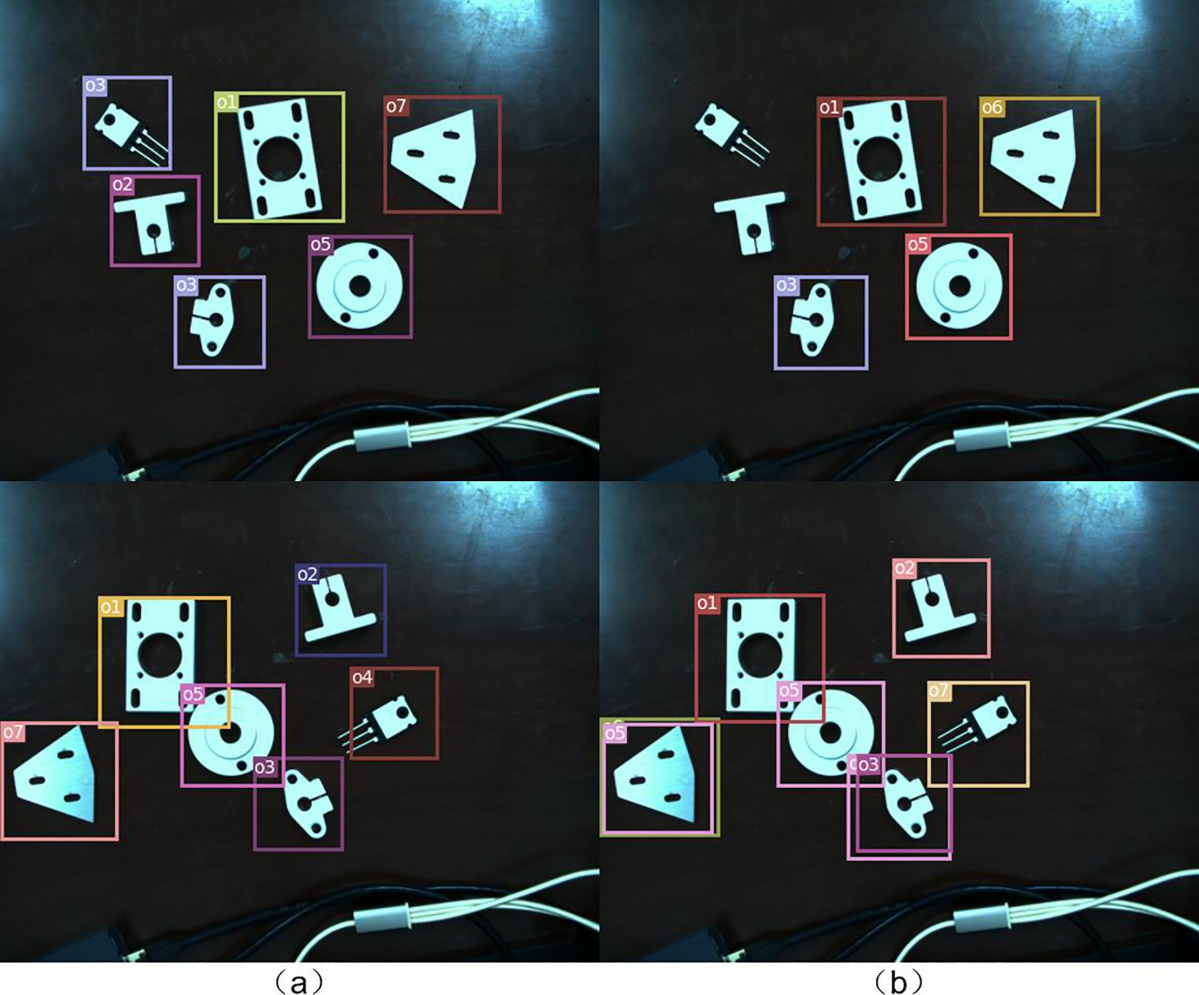
The test result of Den-yolov3 and stand yolov3: (a) Den-yolov3 test result. (b) yolov3 test result.

**Fig 14:**
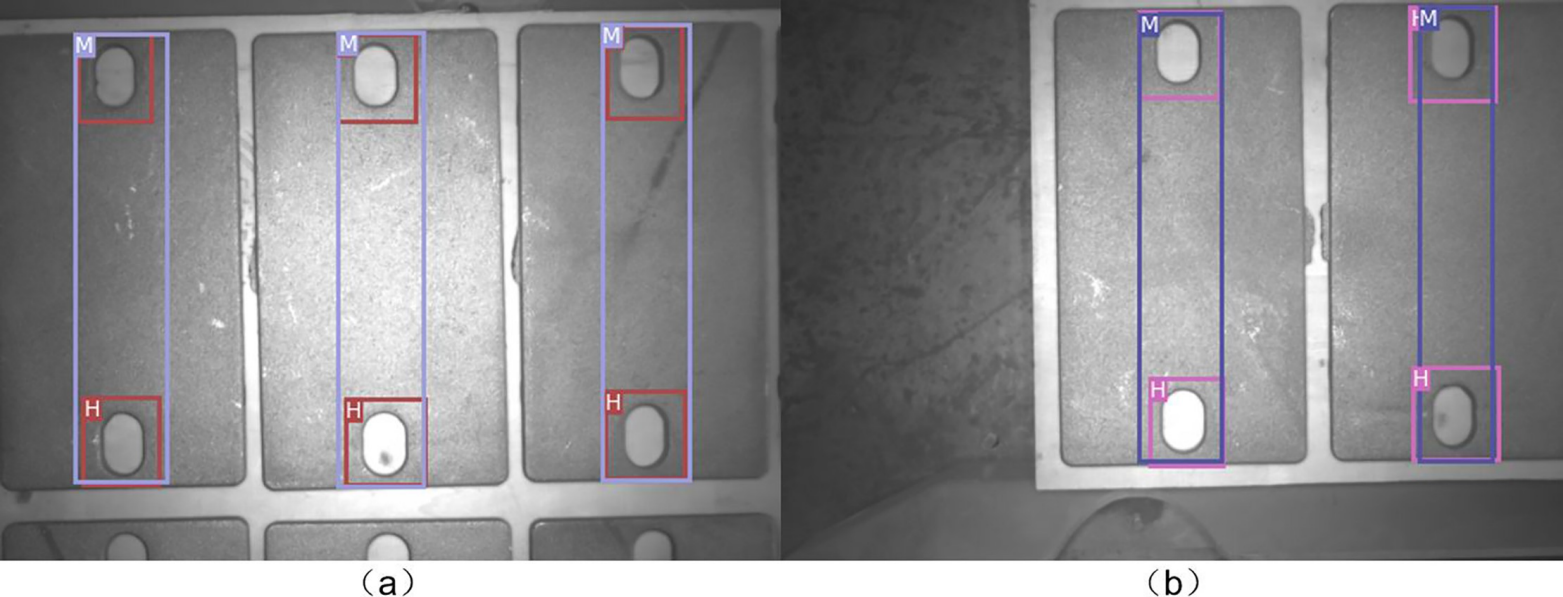
The object in this study test result of Den-yolov3.

It can be seen from the Fig 13 that Den-yolov3 has better recognition effect than yolov3 under the same training times. Fig 14 shows that the detect result of Den-yolov3 to the object in this study.

### Experimental results of fine positioning

The object was placed in a fixed position and the industrial manipulator was used to get 50 images in different positions. The single LINE–2D method and the GMLINE-2D method were used to process the images and record the matching score. Following this, the GMLINE-2D method was used to calculate the object grasp pose for the 70 images. Since the object was fixed, the grasp pose would be similar. The results thus obtained are plotted in Fig 13, and the numerical values are given in Table 6.

**Table 6:** GMLINE-2D calculate result.

images	x/mm	y/mm	c/°
1	362.74	–282.37	92.5
2	363.53	–282.43	92.7
3	363.21	–282.24	92.4
4	362.64	–283.11	93.2
5	362.66	–282.53	92.5
6	362.26	–281.88	92.2
7	361.87	–282.32	92.3
8	363.24	–282.28	92.7
9	361.98	–283.15	93.4
. . .	. . .	. . .	. . .
70	362.18	–283.07	92.4
*average error*	±2	±2	±1

**Fig 15:**
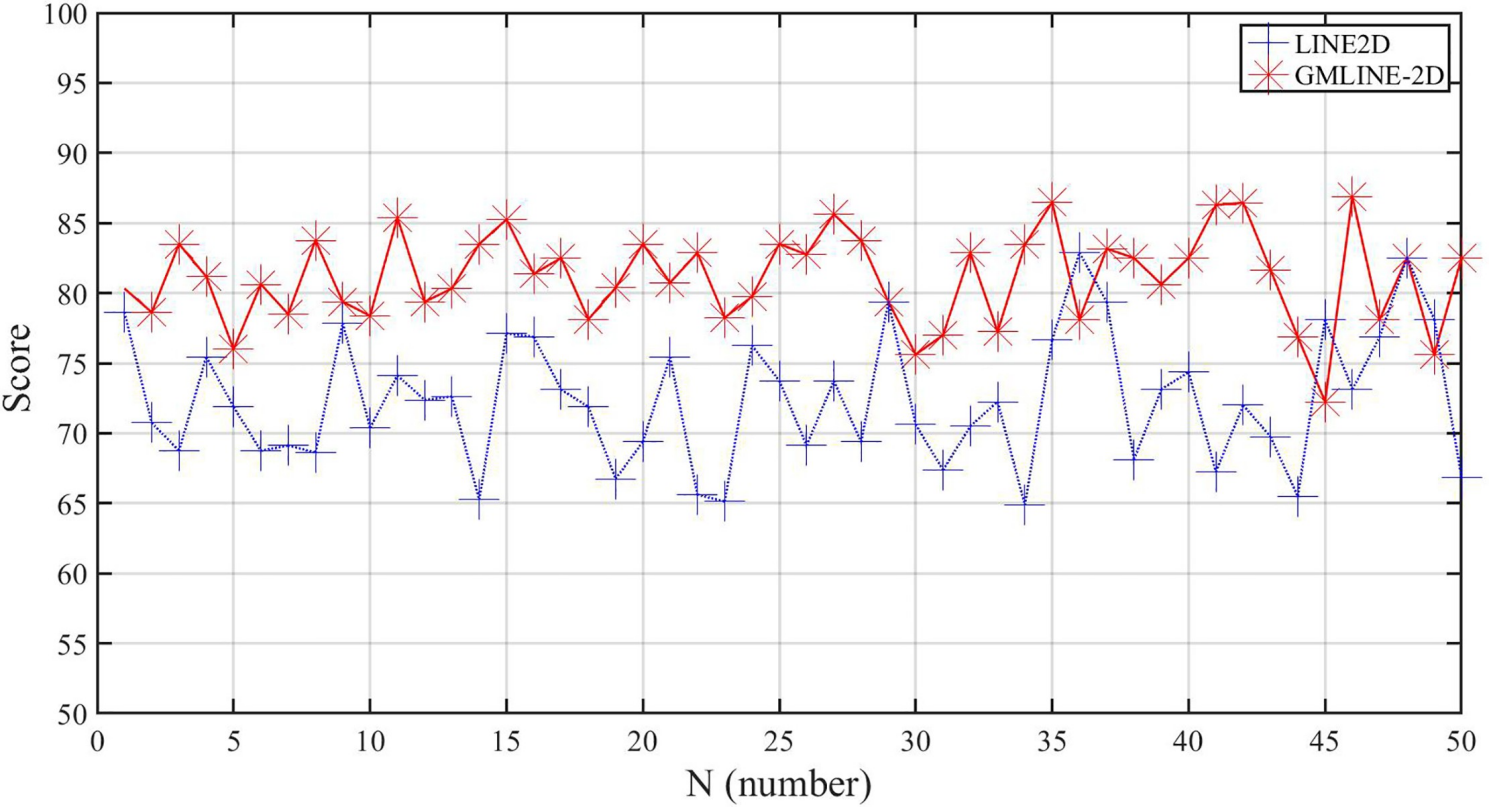
Results of Line2D method result and GMLINE-2D.

From Fig 15, it can be seen that the GMLINE-2D matching score is high and robust as compared to the Line-2D method, which indicates that the GMLINE-2D is more robust. GMLINE-2D uses multiple models to match the local features of the object, and selects the highest similarity score as the final matching result. Compared with Line-2D, which only use one model. Thus, it can obtain better similarity score.

From Table 6, it can be seen that the errors in the X and Y values of the grasp position are less than 2 mm and the c axes grasp value is less than 1°; these errors are sufficiently small for a rough object grasp.

**Fig 16:**
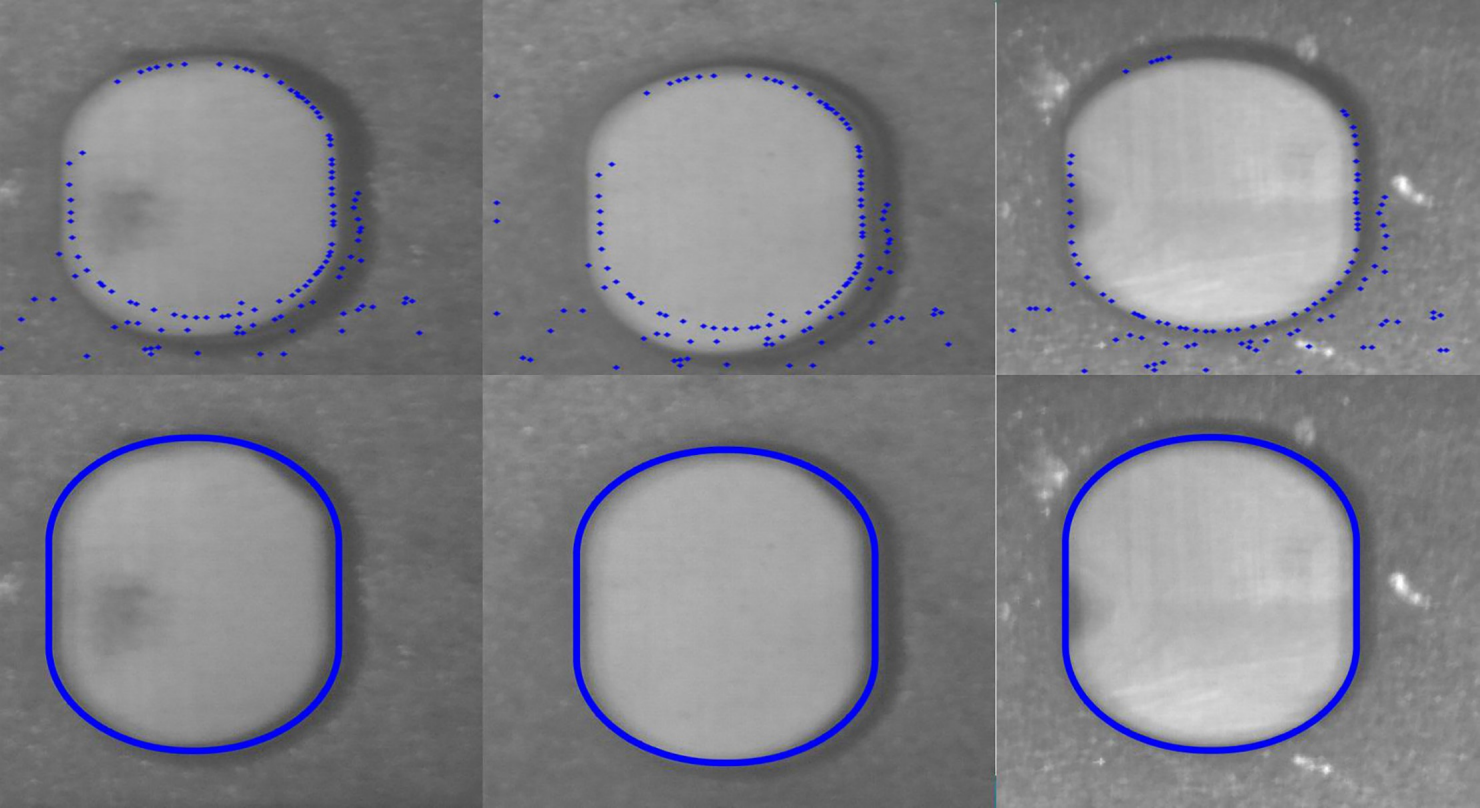
Line-MOD and GMLINE-2D matching result.

Fig 16 shows the matching comparison between Line-MOD and our method. It can be seen from the figure that Line-MOD only uses a small number of feature points on the object surface. The proposed method in this study makes full use of the contour information of the object and has a good matching effect.

**Fig 17:**
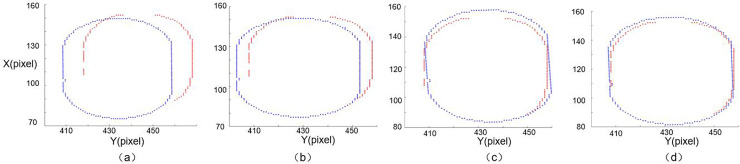
Fast ICP registration for a feature area (X and Y coordinates are in pixels). (a) the point after GMLINE-2D, (b) first time iterative, (c) second time iterative, (d) last time iterative.

Fig 17 is the result of the gradient feature approximation between the template and the input image feature area after several iterations using the ICP algorithm. We choose the area of the hole on the surface of the object as the matching feature. From the registration results, we can find that even if there is a matching error due to interference in the image feature area, the ICP algorithm can still improve the image matching accuracy based on the positioning of the matching algorithm.

**Fig 18:**
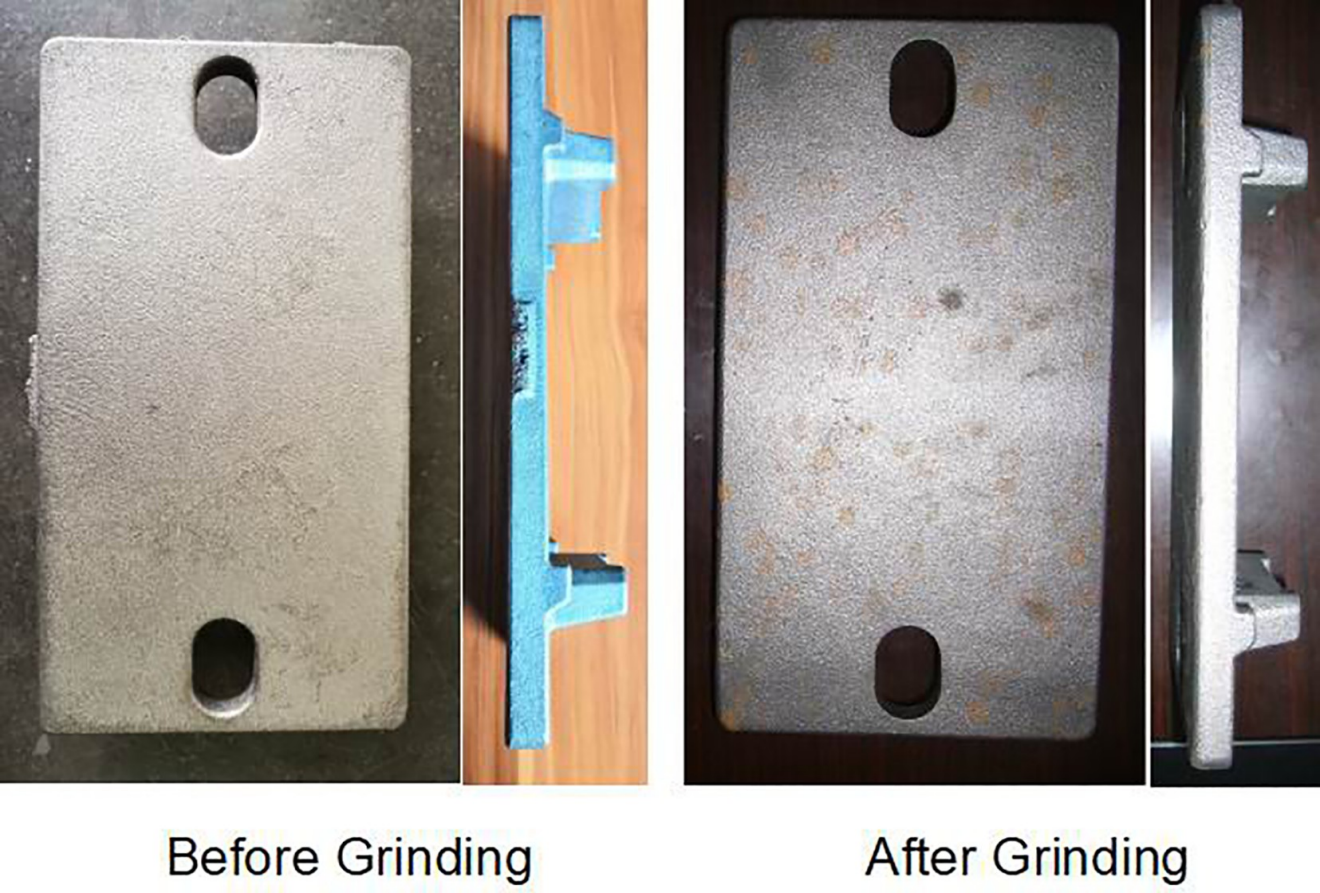
Picture showing the object before (left) and after (right) grinding (the burr on the workpiece surface is cleanly removed).

The grinding station combines the burr detection and trajectory planning functions of the vision system. From the combined applications of visual positioning, RANSAC line fitting, grid burr detection, and coordinate transformation, the automation of the grinding system is greatly improved. On the one hand, the workstation completes the robot positioning and grasping of the rough object under complex background conditions. On the other hand, the system realizes accurate online detection of the burr of the casting nozzle on the surface of the object, and based on the detection result; the industrial manipulator trajectory is generated offline. The grinding process is unmanned, which optimizes the working efficiency of the robot polishing and improves the safety factor of the workstation. A comparison showing the object before and after grinding is shown in Fig 18:

## Conclusion

This paper proposes an industrial manipulator grinding station based on the vision system for robotic grasping guidance and automatic planning of the grinding trajectory. The workstation realizes the automation of the entire process from automatic loading to grinding. A novel coarse-to-fine vision position strategy which consists of an improved deep neural network detector and uses the industrial object recognition along with the modified Line-MOD method for precise positioning, is proposed. This strategy can locate the rough casting position precisely and robustly. This study also proposes a method based on the grid method for industrial manipulator grinding trajectory. By extracting the edge gradient of the object and combining this information with the RANSAC straight line fitting method to obtain the position of the burr, the grinding position can be generated. Combined with the industrial manipulator control logic, the workstation can complete the automated production of a blank object from the pallet to the grinding and placing processes.

The robot grinding station has the advantages of high grasping precision and good consistency in the quality of grinding the workpiece. At the same time, the workstation can complete the work that originally required 3–4 per unit working time for carrying out the process manually. The system also reduces labor during the production and debugging process. The less amount of intervention time required in this setup increases the safety of the staff. In the future, we will further improve the function of the proposed algorithm, further improve the detection accuracy and detection speed of the detection network, and consider adding rotation detection function in the network.

## Supporting information

S1 File (DOC)

## References

[1] Golda G, Kampa A. Modelling of Cutting Force and Robot Load during Machining. Advanced Materials Research, 2014: 715–720.

[2] Fan X, Wang X, Xiao Y, et al. A combined 2D-3D vision system for automatic robot picking. international conference on advanced mechatronic systems, 2014: 513–516.

[3] Pilný L, Bissacco G, De Chiffre L, et al. Acoustic emission-based in-process monitoring of surface generation in robot-assisted polishing. International Journal of Computer Integrated Manufacturing, 2016, 29(11): 1218–1226.

[4] Zhu W., Wang Y., Shen H., et al. Design and experiment of compliant parallel humanoid wrist joint polishing robot. Transactions of the Chinese Society for Agricultural Machinery, 2016.

[5] Qin Z, Wang P, Sun J, et al. Precise Robotic Assembly for Large-Scale Objects Based on Automatic Guidance and Alignment. IEEE Transactions on Instrumentation and Measurement, 2016, 65(6): 1398–1411.

[6] Wang G, Wang Y, Zhang L, et al. Development and Polishing Process of a Mobile Robot Finishing Large Mold Surface. Machining Science and Technology, 2014, 18(4): 603–625.

[7] Park I, Lee I, Lee J, et al. Development of the robot system for the process improvement of the castings of the runner and gate cutting. international conference on ubiquitous robots and ambient intelligence, 2013: 760–762.

[8] Gaz C, Magrini E, De Luca A, et al. A model-based residual approach for human-robot collaboration during manual polishing operations. Mechatronics, 2018: 234–247.

[9] Sornmo O, Robertsson A, Wanner A, et al. Force controlled knife-grinding with industrial robot. international conference on control applications, 2012: 1356–1361.

[10] Du H, Sun Y, Feng D, et al. Automatic robotic polishing on titanium alloy parts with compliant force/position control. Proceedings of the Institution of Mechanical Engineers, Part B: Journal of Engineering Manufacture, 2015, 229(7):1180–1192.

[11] Zhang H, Li L, Zhao J, et al. Robot automation grinding process for nuclear reactor coolant pump based on reverse engineering. The International Journal of Advanced Manufacturing Technology, 2019: 879–891.

[12] Pandiyan V, Murugan P, Tjahjowidodo T, et al. In-process virtual verification of weld seam removal in robotic abrasive belt grinding process using deep learning. Robotics and Computer-integrated Manufacturing, 2019: 477–487.

[13] Gottesfeld Brown L. A survery of image registration techniques. Acm Computing Surveys, 1992, 24(4):325–376.

[14] Ulrich M, Steger C, Baumgartner A, et al. Real-time object recognition using a modified generalized Hough transform. Pattern Recognition, 2003, 36(11): 2557–2570.

[15] Li J, Gu J, Huang Z, et al. Application Research of Improved YOLO V3 Algorithm in PCB Electronic Component Detection. Applied Sciences, 2019, 9(18).

[16] Redmon J, Divvala S K, Girshick R, et al. You Only Look Once: Unified, Real-Time Object Detection. computer vision and pattern recognition, 2016: 779–788.

[17] Redmon, J. and Farhadi, A. YOLO9000: Better, Faster, Stronger. Proceedings of the IEEE Conference on Computer Vision and Pattern Recognition, Honolulu, 21–26 July 2017, 6517–6525.

[18] Redmon J, Farhadi A. YOLOv3: An Incremental Improvement. arXiv: Computer Vision and Pattern Recognition, 2018.

[19] Pang S, Ding T, Qiao S, et al. A novel YOLOv3-arch model for identifying cholelithiasis and classifying gallstones on CT images. PLoS ONE, 2019, 14(6):e0217647–. 10.1371/journal.pone.0217647 31211791 PMC6581241

[20] Chen Y, Li W, Sakaridis C, et al. Domain Adaptive Faster R-CNN for Object Detection in the Wild. computer vision and pattern recognition, 2018: 3339–3348.

[21] Ghiasi G, Lin T, Le Q V, et al. NAS-FPN: Learning Scalable Feature Pyramid Architecture for Object Detection. computer vision and pattern recognition, 2019: 7036–7045.

[22] Hinterstoisser S, Cagniart C, Ilic S, et al. Gradient Response Maps for Real-Time Detection of Textureless Objects. Pattern Analysis and Machine Intelligence, IEEE Transactions on, 2012, 34(5):p.876–888. 10.1109/TPAMI.2011.206 22442120

[23] Hinterstoisser S, Lepetit V, Ilic S, et al. Dominant orientation templates for real-time detection of texture-less objects. computer vision and pattern recognition, 2010: 2257–2264.

[24] Hinterstoisser S, Holzer S J, Cagniart C, et al. Multimodal templates for real-time detection of texture-less objects in heavily cluttered scenes. international conference on computer vision, 2011: 858–865.

[25] Zhang Z. A flexible new technique for camera calibration. IEEE Transactions on Pattern Analysis and Machine Intelligence, 2000. 22(11), 1330–1334.

