# Supplementary material for: A Robotic grinding station based on an industrial manipulator and vision system
Source: PLoS One. 2021 Mar 24;16(3):e0248993. doi: 10.1371/journal.pone.0248993 (PMC7990196; doi:10.1371/journal.pone.0248993)
Supplement: S1 File — (DOC) [file pone.0248993.s001.doc]

We upload the dataset we create to figshare, (DOI:10.6084m9.figshare.13256909)

Limited by the storage space, we did not upload all the data, but we think the uploaded data will not affect the test. If the reviewer or reader needs more comprehensive data information, please contact us.
